# Doxycycline Stabilizes Vulnerable Plaque via Inhibiting Matrix Metalloproteinases and Attenuating Inflammation in Rabbits

**DOI:** 10.1371/journal.pone.0039695

**Published:** 2012-06-21

**Authors:** Mei Dong, Lin Zhong, Wen Qiang Chen, Xiao Ping Ji, Mei Zhang, Yu Xia Zhao, Li Li, Gui Hua Yao, Peng Fei Zhang, Cheng Zhang, Lei Zhang, Yun Zhang

**Affiliations:** 1 The Key Laboratory of Cardiovascular Remodeling and Function Research, Chinese Ministry of Education and Chinese Ministry of Health, Shandong University Qilu Hospital, Jinan, Shandong, China; 2 Yu Huang Ding Hospital, Yantai, Shandong, China; Brigham and Women's Hospital, Harvard Medical School, United States of America

## Abstract

Enhanced matrix metalloproteinases (MMPs) activity is implicated in the process of atherosclerotic plaque instability. We hypothesized that doxycycline, a broad MMPs inhibitor, was as effective as simvastatin in reducing the incidence of plaque disruption. Thirty rabbits underwent aortic balloon injury and were fed a high-fat diet for 20 weeks. At the end of week 8, the rabbits were divided into three groups for 12-week treatment: a doxycycline-treated group that received oral doxycycline at a dose of 10 mg/kg/d, a simvastatin-treated group that received oral simvastatin at a dose of 5 mg/kg/d, and a control group that received no treatment. At the end of week 20, pharmacological triggering was performed to induce plaque rupture. Biochemical, ultrasonographic, pathologic, immunohistochemical and mRNA expression studies were performed. The results showed that oral administration of doxycycline resulted in a significant increase in the thickness of the fibrous cap of the aortic plaque whereas there was a substantial reduction of MMPs expression, local and systemic inflammation, and aortic plaque vulnerability. The incidence of plaque rupture with either treatment (0% for both) was significantly lower than that for controls (56.0%, P<0.05). There was no significant difference between doxycycline-treated group and simvastatin-treated group in any serological, ultrasonographic, pathologic, immunohistochemical and mRNA expression measurement except for the serum lipid levels that were higher with doxycycline than with simvastatin treatment. In conclusion, doxycycline at a common antimicrobial dose stabilizes atherosclerotic lesions via inhibiting matrix metalloproteinases and attenuating inflammation in a rabbit model of vulnerable plaque. These effects were similar to a large dose of simvastatin and independent of serum lipid levels.

## Introduction

Atherosclerosis is a complex inflammatory and proliferative process and plaques vulnerable to rupture, the major cause of acute coronary syndrome, are characterized by an atrophic fibrous cap, a lipid-rich necrotic core, accumulation of inflammatory cells such as monocytes/macrophages, and imbalance between extracellular matrix synthesis and degradation [1], [2]. Therefore, the major determinants of plaque vulnerability are progressive lipid accumulation (core formation) and fibrous cap weakening due to ongoing inflammation with collagen degradation (macrophages-related) and impaired healing and repair [vascular smooth muscle cells (VSMCs)-related].

A wealth of evidence has pointed to matrix metalloproteinases (MMPs) as a major molecular mediator of plaque vulnerability [3], [4]. MMPs are a group of more than 20 zinc-containing endopeptidases that are secreted or expressed at the cell surface of all main vascular cell types. Members of the MMPs family include collagenases (MMP-1, MMP-8 and MMP-13), gelatinases (MMP-2 and MMP-9), stromelysins (MMP-3, MMP-10 and MMP-11), matrilysins (MMP-7) and membrane-type MMPs, and each of these MMPs can process at least one type of extracellular matrix (ECM). MMPs have overlapping specificities, but specific MMPs may have different and even contradicting roles in the natural history of atherosclerosis. Pathological findings have implicated MMPs in all stages of atherosclerosis, from lesion formation to plaque progression. A role of MMPs in atherogenesis may be inferred by the detection of MMP-1, MMP-2, MMP-3, MMP-9 and MMP-12 in atherosclerotic lesions [3], [4]. In the early stage of atherosclerosis, MMPs may facilitate migration of VSMCs and monocytes/macrophages, and thus enhance plaque formation. In the late stage of atherosclerosis, accumulated MMPs may degrade the fibrous cap and lead to plaque disruption and atherothrombosis, causing acute coronary syndromes. Thus, MMPs might offer an attractive therapeutic target for plaque attenuation and stabilization. One approach to the inhibition of MMPs is to use pharmacological inhibitors. Although statins are effective in inhibiting MMPs expression in atherosclerotic lesions, it may cause side effects such as liver dysfunction and myopathy in some patients. Recent studies found that doxycycline, a well-known antibiotic drug, may exert powerful inhibitory effects on MMPs activity. However, in different experimental and clinical studies, doxycycline treatment exhibited diverse or even opposite impacts on plaque stability and patient outcome, making such a therapy inconclusive [5], [6], [7]. In the present study, we hypothesized that doxycycline was as effective as simvastatin in reducing the incidence of plaque disruption via inhibiting MMPs activity and attenuating local inflammation. A series of experiments *in vivo* and *in vitro* were designed and performed to test this hypothesis.

**Table 1:** Primers for RT-PCR.

molecules	loci	primer sequence
GAPDH	NM_001082253	S: GAGCTGAACGGGAAACTCAC
		A: GGTCTGGGATGGAAACTGTG
p-selectin	NM_001082755	S: CTGACAATGAGCCTAACAACAAG
		A: CTATAGCAGAGTGCTCGCTTTC
MCP-1	NM_001082294	S: CAGCCAGATGCCGTGAA
		A: TTGGGTTGTGGAATAAGAGGT
MMP-1	NM_001082793	S: GAGGAGGAGACGGAGGTGAT
		A: GGGAACGCTGGCAGTAGAG
MMP-2	NM_001082209	S: GTCTGAAGAGCGTGAAGGTTGG
		A: GTTGACGGGATTGGAGGGGAAG
MMP-3	NM_001082280	S: CGCTTTGCTCAGCCTATCCAC
		A: CCAACATCAGGAACGCCACA
MMP-9	NM_001082203	S: CGGAGCACGGAGACGGGTAT
		A: GATGAAGGGGAAGTGGCAGG
MMP-12	NM_001082771	S: GGCACAAACTTGTTCCTTGTTG
		A: TGGGTTGATGCTGCTCTGG

GAPDH: glyceraldehydes 3-phosphate dehydrogenase; MCP-1: monocyte chemoattractant protein-1; MMP-1: matrix metalloproteinase 1; MMP-2: matrix metalloproteinase 2; MMP-3: matrix metalloproteinase 3; MMP-9: matrix metalloproteinase 9; MMP-12: matrix metalloproteinase 12.

## Materials and Methods

### Ethics Statement

The experiments complied with the Animal Management Rule of the Chinese Ministry of Health (documentation 55, 2001), and the experimental protocol was approved by the Animal Care Committee of Shandong University.

**Figure 1:**
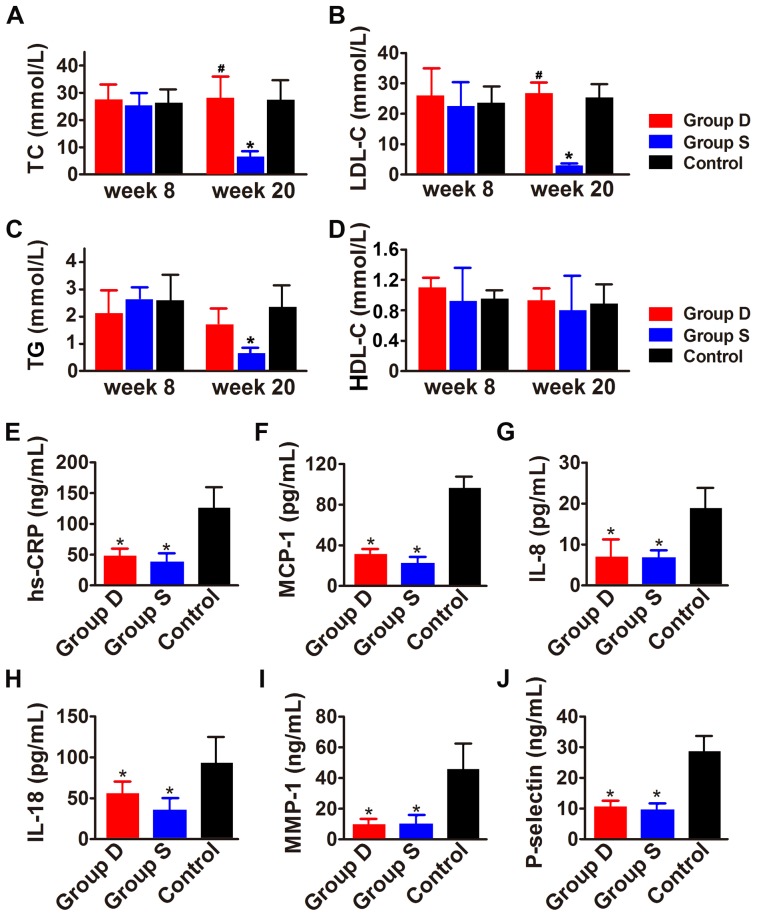
Biochemical measurements in three groups of rabbits. Panel A, B, C and D show the serum levels of TC, LDL-C, TG and HDL-C, respectively, in three groups of rabbits at week 8 and week 20. Panel E, F, G H, I and J depict the serum levels of hs-CRP, MCP-1, IL-8, IL-18, MMP-1 and P-selectin, respectively, in three groups of rabbits at week 20. Group D: doxycycline-treated group; Group S: simvastatin-treated group; Control: control group. *P<0.05 vs. Control group; ^#^ *P*<0.05 vs. Group S.

### Animal protocol

A total of 30 male New Zealand White rabbits weighing 2–3 kg were housed at the Animal Care Center of Shandong University Qilu Hospital. All rabbits underwent balloon-induced endothelial injury of the abdominal aorta and received a high-fat diet (1% cholesterol, Shandong Experimental Animal Center, Jinan, China) feeding for 20 weeks. Balloon-induced aortic wall injury was performed with a 4-F balloon catheter (balloon size of 3.5 mm in diameter and 15 mm in length) introduced through the right femoral artery to the thoracic aorta after anesthetization. The balloon was inflated with saline to increase the pressure to 8 atm and the catheter was retracted down to the iliofemoral artery. This process was repeated three times in each rabbit to ensure denudation of the endothelium of the abdominal aorta. From the end of week 8 to the end of week 20, rabbits were randomly divided into three groups (n = 10 each): doxycycline-treated group that received doxycycline (Chemical material plant, Jiangsu, China) at an oral dose of 10 mg/kg/d [8], simvastatin-treated group that received simvastatin (Merck & Co. Inc, Hangzhou, China) at an oral dose of 5 mg/kg/d, and control group that received no treatment. These drugs were supplemented in water and administered by oral gavage. At the end of week 20, all rabbits underwent pharmacological triggering as described previously [9], [10]. In brief, 0.15 mg/kg of Chinese Russell's viper venom was injected intraperitoneally, followed 30 min later by an intravenous injection of 0.02 mg/kg histamine (Sigma, St. Louis, MO, USA). High-frequency ultrasonography and intravascular ultrasound imaging were performed in all rabbits before pharmacological triggering to examine the morphological changes of the aortic plaques. Rabbits were euthanized 24 hr after pharmacological triggering by intravenous injection of an overdose of pentobarbital.

**Figure 2:**
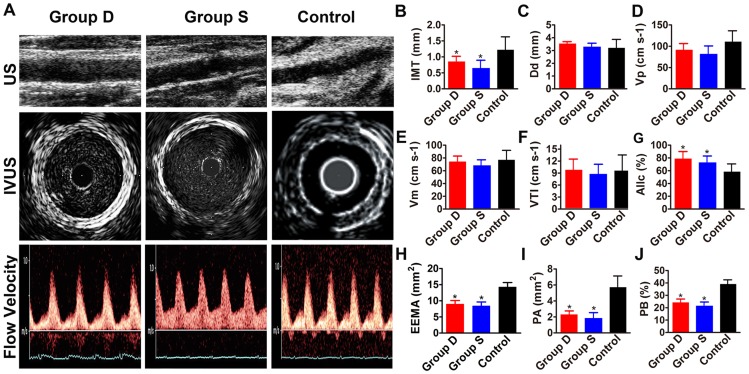
Ultrasonographic and intravascular ultrasound images and measurements in three groups of rabbits. Panel A shows two-dimensional ultrasonographic and intravascular ultrasound (IVUS) images and blood flow velocities of the abdominal aorta in three groups of rabbits. Panel B, C, D, E, F and G depict the measurements of IMT, Dd, Vp, Vm, VTI and AIIc in three groups of rabbits before pharmacological triggering. Panel H, I and J show the measurements of EEMA, PA and PB% in three groups of rabbits before pharmacological triggering. Group D: doxycycline-treated group; Group S: simvastatin-treated group; Control: control group.*P<0.05 vs. Control group.

### Measurement of doxycycline plasma concentration

Blood samples were collected from all rabbits of doxycycline-treated group, and the plasma concentration of doxycycline was monitored by high-performance liquid chromatography (HPLC) [11] by use of a Waters 515 HPLC instrument at day 1 and week 1, 4, 8 and 12 after doxycycline administration. Separation was performed on a Waters analytical column (4.6×250 mm, 5 µm), with the mobile phase consisting of acetonitrile and water with gradient elution at a flow rate of 0.8 ml/min and a column temperature of 30°C. The UV wavelength used for detection was 347 nm and the analysis time 6.195 min.

The standard curve of plasma concentration of doxycycline was identified on the HPLC chromatogram and individual plasma concentrations of doxycycline were calculated from the regression equation obtained from 7 standard concentrations (0.1, 0.25, 0.5, 1.0, 2.0, 4.0, and 8.0 µg/ml).

**Figure 3:**
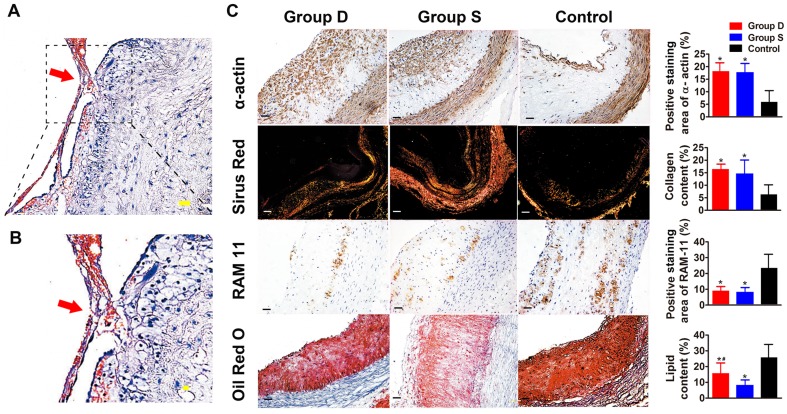
Immunohistochemical staining of plaque components in three groups of rabbits. Panel A and B depict the H&E staining of the abdominal aortic cross-section in a rabbit of the control group, showing an intraluminal thrombus attached to a disrupted plaque (Bars = 100 µm). Panel C show dense α-actin of smooth muscle cells in the doxycycline-treated group (Group D) and simvastatin-treated group (Group S) and sparse α-actin of smooth muscle cells in the Control group. Sirius-red staining illustrates abundant collagen in Group D and Group S and less collagen in the Control group. RAM 11 staining demonstrates few macrophages in Group D and Group S and ample macrophages in the Control group. Oil-red O staining shows a small, moderate and large amount of lipids in Group S, Group D, and Control group, respectively. (Bars = 100 µm) Group D: doxycycline-treated group; Group S: simvastatin-treated group; Control: control group. *P<0.05 vs. Control group; *^#^ *P*<0.05 vs. Control group and *P*<0.05 vs. Group S.

### Biochemical studies

In all rabbits, blood samples were collected at the beginning of the experiment and before pharmacological triggering. Serum levels of total cholesterol (TC), triglycerides (TG), high-density lipoprotein cholesterol (HDL-C) and low-density lipoprotein cholesterol (LDL-C) were measured by enzymatic assays. Serum levels of high sensitive C-reactive protein (hs-CRP), monocyte chemoattractant protein-1 (MCP-1), interleukin (IL)-8, IL-18, MMP-1 and P-selectin were assayed by use of ELISA kits (R&D Systems, Chicago, IL, USA).

**Figure 4:**
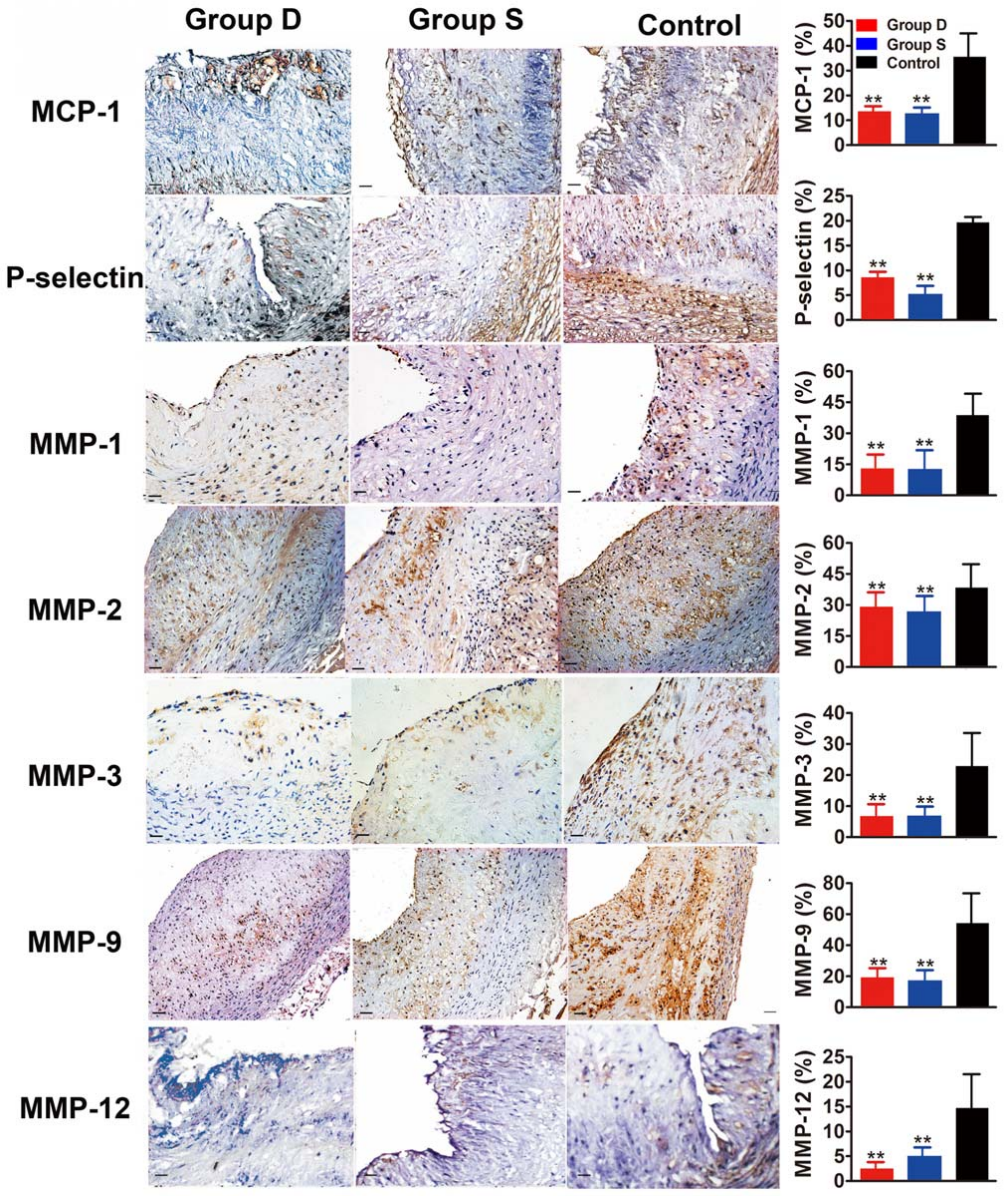
Immunohistochemical staining of the inflammatory markers in the aortic plaque in three groups of rabbits. The left panel shows protein expression of MCP-1, P-selectin, MMP-1, MMP-2, MMP-3, MMP-9 and MMP-12. The right panel indicates the quantitative analysis of the results in the left panel. Bars = 50 µm. Group D: doxycycline-treated group; Group S: simvastatin-treated group; Control: control group. *P<0.05 vs. Control group; ** *P*<0.01 vs. Control group.

### Ultrasonographic Study

#### High-frequency ultrasonography

A high-frequency duplex ultrasonographic system (HP SONOS 5500, Andover, Massachusetts, USA) connected with a 7.5-MHz transducer were applied to detect the aortic plaques before pharmacological triggering. The aortic diameter at end-diastole (Dd) and the maximal intima-media thickness (IMT) were measured and the aortic peak velocity (Vp), mean velocity (Vm) and velocity-time integral (VTI) were recorded.

**Figure 5:**
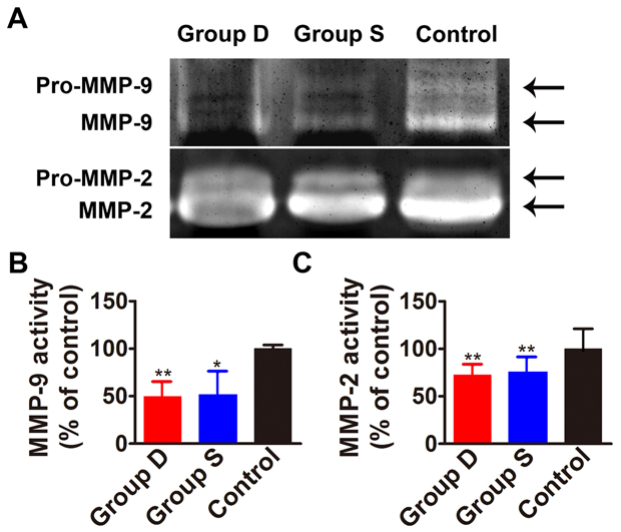
Enzymatic activity of MMP-2 and MMP-9 in the aortic plaque in three groups of rabbits. Panel A shows a representative zymogram of MMP-2 and MMP-9. Panel B and C depicts the quantitative analysis of the results in panel A. Group D: doxycycline-treated group; Group S: simvastatin-treated group; Control: control group. *P<0.05 vs. Control group; ** *P*<0.01 vs. Control group.

#### Integrated backscatter analysis

The acoustic densitometry technique was applied to analyze the ultrasonic integrated backscatters from the aortic wall and plaques. The ultrasonic intensity (AII) of the aortic intima and adventitia in normal segments and aortic plaques were recorded, and the corrected AII (AIIc%) was calculated as the ratio of AII of the intima to that of the adventitia in both normal segments and plaques.

**Figure 6:**
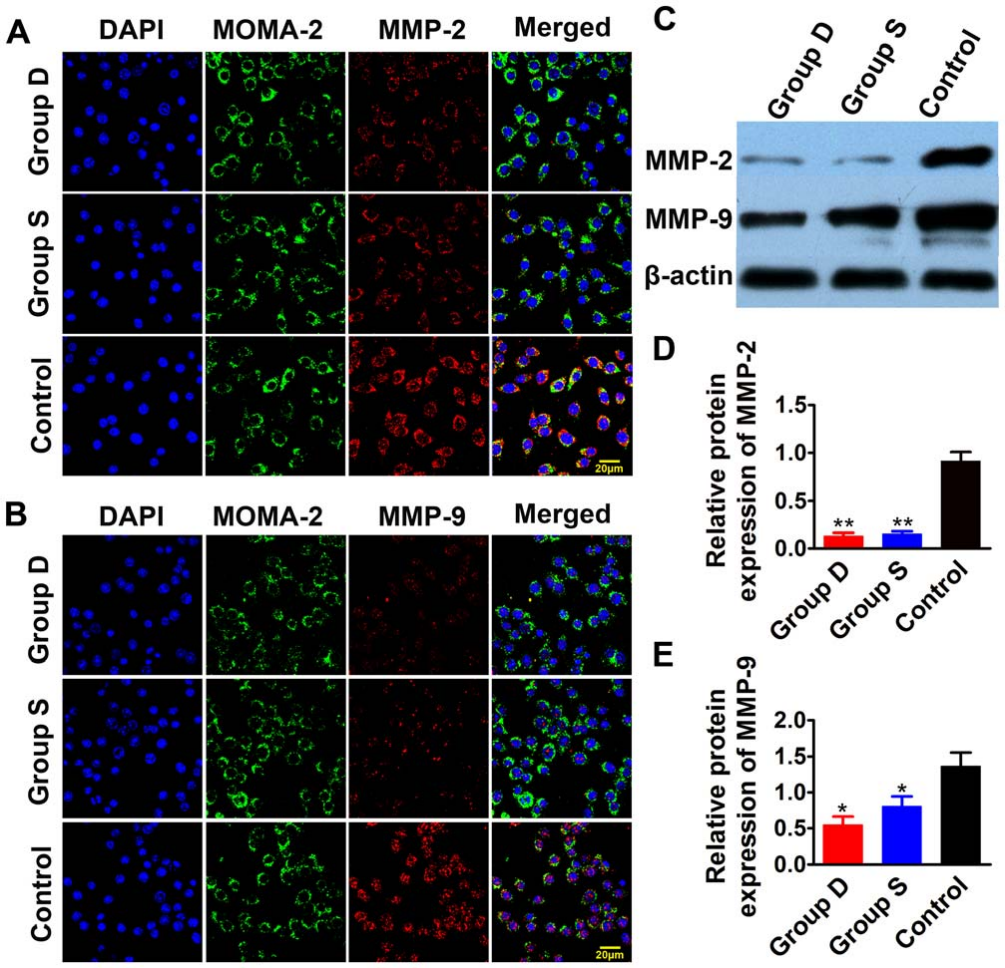
Immunofluorescence and western blot analysis of MMP-2 and MMP-9 expression *in vitro*. Panel A shows the immunofluorescence images of MMP-2 in macrophages receiving doxycycline, simvastatin or no treatment. Panel B shows the immunofluorescence images of MMP-9 in macrophages receiving doxycycline, simvastatin or no treatment. Panel C shows western blot analysis of MMP-2 and MMP-9 expression. Panel D and E are the quantitative analysis of Panel C. Blue color represents DAPI staining, green color MOMA-2 staining and red color MMP-2 or MMP-9 staining. Bars = 20 µm. Group D: doxycycline-treated group; Group S: simvastatin-treated group; Control: control group. **P<0.01, vs. Control group.

#### Intravascular ultrasound (IVUS) imaging

IVUS imaging was performed before pharmacological triggering using a 3.2 F catheter that contains a single rotating element transducer of 40 MHz connected to an IVUS system (Galaxy, Boston Scientific Corp., Fremont, CA, USA). The catheter was withdrawn from the aortic arch to the abdominal aorta with a motorized pullback device at a constant speed of 0.5 mm/s. The external elastic membrane area (EEMA) and lumen area (LA) were measured to calculate the plaque area (PA) as: PA = EEMA-LA, and plaque burden was then derived using the formula: PB% = PA/EEMA×100%.

### Histopathology and immunohistochemistry

The abdominal aorta was examined to observe the incidence of plaque rupture and thrombosis. Tissue samples 2 cm long were taken from the abdominal aorta and fixed in 4% formaldehyde. Some segments were embedded in paraffin and cut into 5-µm-thick sections for staining with hematoxylin and eosin (H&E) and Masson trichrome, whereas other sections were stained with sirius red and Oil-red O (Santa Cruz Biotechnology, Santa Cruz, CA, USA) and reacted with mouse anti-rabbit MMP-3 monoclonal antibody (Oncogene, USA), mouse anti-rabbit RAM-11 monoclonal antibody (Dako, USA), mouse anti-rabbit α-smooth-muscle-cell (SMC) actin monoclonal antibody (Sigma Chemical, USA), MMP-1, MMP-2 (Abcam, Cambridge, MA, USA), MMP-3, MMP-9 (Chemicon, Boston, MA, USA), MMP-12, P-selectin and MCP-1 polyclonal antibody (Santa Cruz Biotechnology). Sections reacted with non-immune IgG, secondary antibody only and no primary and secondary antibodies were used as negative controls.

Histopathological slides were analyzed by use of a computer-assisted morphometric analysis system (Image-Pro Plus 5.0, Media Cybernetics, Cambridge, MA, USA). The fibrous cap thickness and IMT of the aortic plaque were measured at 10 equidistant points around the cap in each slice; three slices per segment were measured and the values were averaged. Then, the ratio of the fibrous cap thickness to IMT was calculated. The positive staining area of α-actin (SMCs), sirius red (collagens), Oil-red O (lipids) and RAM-11 (macrophages) was expressed as a percentage of stained area divided by the plaque area of the aortic plaque in at least 10 high-power fields (×400). The vulnerability index (VI) was derived as: VI = (macrophages stained %+lipids stained %)/(smooth muscle cells stained %+collagen stained %) [12]. Plaque rupture was defined as discontinuity of the fibrous cap with luminal thrombosis or a buried fibrous cap within a plaque [13].

### Gelatin zymography

MMP-2 and MMP-9 enzymatic activities were measured by gelatin-zymography. The proteins were extracted from snap-frozen tissue samples of the abdominal aorta and mixted with the same volume of Tris-Glycine SDS Sample Buffer. The protein samples were separated by electrophoresis on a sodium dodecylsulfate–10% polyarylamide gels containing 1 mg/ml gelatin (Sigma, St. Louis, MO, USA) and the gels were rinsed twice in 50 mls of 2.5%(v/v) Triton-X-100 for 30 minutes at room temperature. Then gels were incubated in fresh incubation-solution and moderately agitated for 48 hours at 37°C. The gels were subsequently stained with 2.5% Coomasie brilliant blue R-250 (Sigma) for 2 hours at room temperature. After destaining for about 1.5 hours, the enzymatic activities of the MMP-2 and MMP-9 were displayed as white bands against blue background and calculated by densitometric analysis.

### RT-PCR

Total RNA was extracted from frozen tissue samples of abdominal aortas, and mRNA expression level of MCP-1, MMP-1, MMP-2, MMP-3, MMP-9, MMP-12 and P-selectin in the aortic plaque was quantitated by RT-PCR using LightCycler (Roche Applied Science, Indianapolis, IN, USA) following the manufacturer's instruction. The mRNA sequences were obtained from GenBank (Bethesda, Maryland, USA, Table 1). The transcript amount of glyceraldehyde 3-phosphate dehydrogenase (GAPDH) was taken as an internal RNA control. Quantitative values were obtained from the threshold cycle value (Ct), the point with a significant increase in fluorescence [14]. Experiments were performed in triplicate for each data point.

### Cell Experiment

RAW 264.7 macrophage cell line was used to explore the mechanism of doxycycline induced changes of MMPs and collagen expression. The RAW 264.7 macrophages were seeded in the 6-well plate at 60%–70% confluence, pre-incubated with ox-LDL (100 µg/ml) for 24 hours [15], and divided into doxycycline-treated group (5 µg/ml) [16] and simvastatin-treated group (10 µg/ml) [17] and control group without treatment. Twenty-four hours after treatment, the macrophages were collected for further analysis. For the assessment of mRNA expression, the total RNA was extracted and the mRNA expression levels of MMP-2, MMP-9 and collagen I were quantitated by RT-PCR. To compare the extent of MMPs expression in macrophages, cells were coincubated with MMP-2 and rat anti-mouse monoclonal monocyte/macrophage antibody (MOMA-2, AbD Serotec, Oxford, UK) or with MMP-9 and MOMA-2. The positively stained cells by immunofluorescence were displayed with a confocal microscope (Zeiss-710, Zeiss, Germany) [18]. For quantitative analysis of protein expression levels of MMP2 and MMP9, western blot was performed as described previously [18].

### Statistical analysis

All statistical analyses involved use of SPSS, v11.0 (SPSS Inc, Chicago, IL, USA). Quantitative variables are expressed as means ± SD. Variables with skewed distribution were log-transformed before the *t* test. For continuous variables, differences between two groups were assessed by unpaired Student's t test, and comparison of multiple groups involved the use of ANOVA. Categorical data were analyzed with Kruskal-Wallis and chi-squared tests. A two-tailed *P*<0.05 was considered statistically significant.

## Results

Administration of doxycycline was tolerated well by all rabbits, with no adverse effects being observed. Only one rabbit in the control group died of diarrhea at week 12.

### Plasma concentration of doxycycline

The doxycycline plasma levels measured in rabbits corresponded well with those found in the literature. The plasma concentration of doxycycline in rabbits at day 1 after treatment was 1.28±0.34 µg/ml, which was an effective plasma drug concentration, and no significant differences were detected among plasma concentrations at day 1, week 1, week 4, week 8 and week 12 after doxycycline administration (2.11±0.43, 2.06±0.35, 2.16±0.39, 2.11±0.43 and 2.32±0.36 µg/ml, respectively, P>0.05).

### Serum lipid profile and inflammatory markers

To determine whether doxycycline administration has an effect on lipid metabolism, serum cholesterol and lipoprotein cholesterol concentrations were measured. At the beginning of the experiment, serum lipid levels did not differ among the 3 groups of rabbits (Fig. 1 A, B, C, D). At week 20, simvastatin-treated group showed a significant reduction in serum TC and LDL-C levels as compared with the doxycycline-treated group and the control group (both *P*<0.05), with no significant difference in serum levels of TC, TG, HDL-C and LDL-C between doxycycline-treated and control groups (Fig. 1 A, B, C, D). The serum levels of MCP-1, hs-CRP, IL-8, IL-18, MMP-1 and P-selectin were substantially lower in the two treatment groups than in the control group, with no significant difference in these cytokines between the two treatment groups (Fig. 1 E, F, G, H, I, J).

### High-frequency ultrasonographic measurements

IMT in the aortic plaque was significantly lower in the two treatment groups than in the control group (both *P*<0.05), with no significant difference between doxycycline-treated and simvastatin-treated groups. In contrast, AIIc% was significantly higher in the two treatment groups than in the control group (both *P*<0.05). The values of Dd, Vp, Vm and VTI did not differ among the three groups of rabbits (Fig. 2 A, B, C, D, E, F, G).

### Intravascular ultrasound measurements

Values of EEMA, PA and PB% in the abdominal aorta in the two treatment groups were lower than those in the control group (all *P*<0.05), with no significant difference between the two treatment groups (Fig. 2 H, I, J). However, values of LA did not differ among the three groups of rabbits.

### Histopathological and immunohistochemical analysis

Doxycycline-treated (257±62 µm) and simvastatin-treated (248±57 µm) groups showed a thicker fibrous cap of the aortic plaque than the control group (113±45 µm, both *P*<0.05). In contrast, doxycycline-treated (680±203 µm) and simvastatin-treated (597±159 µm) groups showed a significantly thinner IMT of the aortic plaque than the control group (972±254 µm, both *P*<0.05). As a result, the ratio of the fibrous cap thickness to IMT of the aortic plaque was significantly larger in doxycycline-treated (0.38±0.03) and simvastatin-treated (0.47±0.05) groups than in the control group (0.16±0.04, both *P*<0.05). After pharmacological triggering, none of the rabbits in the doxycycline-treated and simvastatin-treated groups exhibited abdominal aortic plaque rupture whereas 5 rabbits in the control group (5/9, 56%) showed abdominal aortic plaque rupture, with the incidence of plaque disruption significantly higher in the control group than in the two treatment groups (both *P*<0.05, Fig. 3A, B).

In comparison with the control group, doxycycline-treated and simvastatin-treated groups showed increased positive staining area of α-actin (both *P*<0.05) and sirius red (both *P*<0.05) in the abdominal aorta (Fig. 3C). In contrast, doxycycline-treated and simvastatin-treated groups showed decreased positive staining area of RAM-11 (both *P*<0.05) and Oil-red O (both *P*<0.05) in the abdominal aortic segments in comparison with the control group, despite the fact that positive staining area of Oil-red O was less in the simvastatin-treated group than in the doxycycline-treated group (P<0.05) (Fig. 3C). As a result, the plaque vulnerability index in doxycycline-treated (0.72±0.08%) and simvastatin-treated (0.58±0.09%) groups was significantly lower than that in the control group (3.79±0.32%, both *P*<0.05). The percentage of cells positively stained for MCP-1, MMP-1, MMP-2, MMP-3, MMP-9, MMP-12 and P-selectin in the abdominal aortic sections was remarkably lower in doxycycline-treated and simvastatin-treated groups than that in the control group (all *P*<0.05), with no significant difference between the two treatment groups (Fig. 4).

### Enzymatic activities of MMP-2 and MMP-9

The enzymatic activities of MMP-2 and MMP-9 in the aortic plaque were remarkably reduced in the doxycycline-treated and simvastatin-treated (*P*<0.01∼0.05) groups compared to the control group, with no significant difference between the two treatment groups (Fig. 5 A, B, C).

### RT-PCR

The mRNA expression of MCP-1, MMP-1, MMP-2, MMP-3, MMP-9, MMP-12 and P-selectin in the aortic plaques was remarkably lower in the doxycycline-treated and simvastatin-treated groups than in the control group (all *P*<0.05), with no significant difference between the two treatment groups (Fig. S1). Similarly, the mRNA expression of MMP2 and MMP9 in macrophages was markedly lower in the doxycycline-treated and simvastatin-treated groups than the control group (P<0.01∼0.05), with no significant difference between the two treatment groups (Fig. S2A, B). The mRNA expression of collagen I in macrophages was significantly higher in the doxycycline-treated and simvastatin-treated groups than the control group (P<0.001), with no significant difference between the two treatment groups (Fig. S2C).

### Immunofluorescence and western blot analysis *in vitro*

MMP-2 and MMP-9 were profoundly stained by immunofluorescence in macrophages receiving no treatment as compared with those treated by doxycycline and simvastatin (Fig. 6A, B). The protein expression levels of MMP-2 and MMP-9 measured by western blot were markedly lower in the doxycycline-treated and simvastatin-treated groups than the control group (P<0.01∼0.05), with no significant difference between the two treatment groups (Fig. 6C, D, E).

## Discussion

The most important finding of the present study was that in a rabbit model of vulnerable aortic plaque, oral administration of doxycycline markedly inhibited MMPs expression and activities, local and systemic inflammation, as well as aortic plaque vulnerability, which was independent of serum lipid levels. These effects led to a successful prevention of plaque disruption, similar to the effects of a large dose of simvastatin [10]. To the best of our knowledge, our study is the first to report the salutary effects of doxycycline in an animal model of vulnerable plaque.

A major obstacle in evaluating the plaque-stabilizing effects of interventions is the lack of ideal animal model of vulnerable plaque. Pathological studies have identified certain characteristics of human atherosclerotic plaques associated with plaque instability and rupture: increased necrotic core size and macrophage infiltration, and decreased smooth muscle cells and collagen content. These pathological features were observed in the abdominal aortic plaques of rabbits receiving endothelial injury, high-fat feeding and pharmacological triggering as in the present study. Furthermore, the abdominal aorta in rabbits offers an optimal site for balloon endothelial injury and ultrasound imaging. Thus, our animal model provides a useful tool for assessing the therapeutic effects of doxycycline on plaque stability.

Plaque disruption is associated with increased inflammation and destruction of the extracellular matrix within the plaque, and the fibrous cap overlying a necrotic core undergoes catastrophic mechanical breakdown. The finding that mRNA, protein and activity levels of MMPs were increased in vulnerable plaques, particularly at the shoulder of the fibrous cap, has led to the notion that a broad-spectrum and/or high activity of MMPs, especially associated with inflammation, may contribute to pathological plaque matrix destruction, including fibrillar collagens [3], [4]. By this mechanism, MMPs may promote plaque destabilization and disruption, the main cause of acute coronary syndromes in human. Thus, MMPs represent a potential therapeutic target for plaque stabilization.

Recent studies have demonstrated that doxycycline, a tetracycline derivative, is a non-specific broad-spectrum and potent inhibitor of MMPs. Using chemically modified tetracyclines, it has been confirmed that the antibiotic and anti-MMPs activities lie in different regions of the molecule. This family of antibiotics can inhibit the activity of secreted MMPs because they bind the calcium and zinc necessary for maintenance of proper conformation of the MMPs [19], [20]. A number of studies have been performed to examine the effects of doxycycline in atherosclerosis but the results are controversial. Manning MW et al. found that doxycycline had no effect on the extent of atherosclerosis in saline- or AngII-infused mice. In contrast, doxycycline markedly reduced the incidence and severity of abdominal aortic aneurysm [7]. Recently, Sheth RA et al showed that doxycycline dose-dependently inhibited MMPs activities measured by optical molecular imaging in a mouse model of abdominal aortic aneurysm [21]. Ohshima S, et al presented evidence of SPECT molecular imaging that minocycline, another tetracycline derivative, reduced plaque inflammation and stabilized atherosclerotic plaques in rabbits [22]. The MIDAS pilot trial revealed that in patients with acute coronary syndromes, doxycycline treatment significantly reduced the plasma levels of high-sensitivity C-reactive protein [6]. Another clinical trial reported that in patients undergoing carotid endarterectomy, doxycycline treatment significantly reduced the concentration of MMP-1 in carotid plaques due to decreased MMP-1 transcript, but the local concentrations of MMP-2, MMP-3, or MMP-9 were not altered [5]. On the other hand, administration of an oral active MMP-3 inhibitor (CGS 27023A) failed to exert any beneficial effect on atherosclerotic lesions measured in the aortas of LDL receptor knockout mice [23].

In the present study, we found that oral administration of doxycycline for 12 weeks in a rabbit model of vulnerable plaque resulted in a substantial inhibition of MMPs expression, reduction of local and systemic inflammation and enhancement of aortic plaque stability. Several lines of evidence were presented to support our conclusion. First, abdominal ultrasonography and intravascular ultrasound revealed decreased plaque burden, reduced positive vascular remodeling, and increased plaque density in the doxycycline-treated group as compared with the control group. Second, immunohistochemical and molecular biological studies showed a significant decrease in the mRNA and protein expression levels of MCP-1, MMP-1, MMP-2, MMP-3, MMP-9, MMP-12 and P-selectin in the aortic plaques, and a substantial decline in the serum levels of MCP-1, hs-CRP, IL-8, IL-18, MMP-1 and P-selectin after doxycycline treatment. Third, histopathological studies demonstrated a dramatic increase in the fibrous cap thickness and a reduction in IMT and plaque vulnerability index. Finally, no rabbits in the doxycyclin-treated group showed evidence of plaque disruption, indicating a successful prevention of plaque rupture in these rabbits. A notable finding in this study was that different from simvastatin, doxycycline had no effect on serum lipid levels and reduced only moderately the plaque lipid content. Nonetheless, doxycycline therapy markedly inhibited MMPs expression and local inflammation, leading to stable plaque phenotype. The major mechanisms underlying these salutary effects of doxycycline may involve downregualted expression of MMP2 and MMP9 and upregulated expression of collagen I in macrophages. These results lend strong support to the notion that doxycycline treatment is of potent plaque-stabilizing effect which is independent of serum lipid levels, a finding consistent with our previous anti-inflammatory studies [24].

Statins have been recognized as the most potent drugs for stabilizing atherosclerotic plaques mainly through lipid-lowering and anti-inflammation effects. In addition, statins has a strong effect on reducing MMPs protein expression and activities in animal models of atherosclerosis [22]. Thus, we made a head-to-head comparison between simvastatin and doxycycline for their therapeutic effects on vulnerable atherosclerotic plaques, and a high dose (5 mg/kg/d) of simvastatin was used as a comparison standard. There was a great variation in the dosing of doxycycline for rabbits in the literature and we chose the commonly used antimicrobial dose (10 mg/kg/d) of doxycycline for rabbits [8]. We found that apart from the serum levels of TC, LDL-C and TG, which were significantly higher in the doxycycline-treated group than the simvastatin-treated group, all serological, ultrasonographic, pathologic, immunohistochemical and mRNA expression measurements showed no significant difference between the two treatment groups. These results suggested that despite of the lack of lipid-lowering effect, doxycycline was as potent as simvastatin in attenuating ECM degradation and local inflammation, and enhancing plaque stability. In view of the low incidence of side effects of doxycycline, especially at a subantimicrobial dose, this drug may provide a novel approach to the stabilization of vulnerable plaques. Besides, since the mechanisms of doxycycline and simvastatin treatment may involve different signaling pathways, combinatorial administration of the two drugs might offer a synergistic effect on the prevention of plaque disruption.

Several limitations of this study should be mentioned. First, the sample size in the present study was small and our primary conclusion needs confirmation by further studies with a large sample size. Second, although we made careful efforts in choosing the optimal dose of doxycycline, the dose-effect and time-effect of doxycycline therapy was not examined. Third, the detailed molecular mechanisms by which doxycycline inhibited MMPs expression and activity and attenuating plaque inflammation were not explored and further *in vitro* studies were required to dissect possible signaling pathways involving in this novel therapy. Finally, plaque rupture in our animal model may not totally simulate that in patients with acute coronary syndrome and the plaque-stabilizing effect of doxycycline needs to be tested in clinical trial.

In conclusion, doxycycline at a common antimicrobial dose substantially inhibited MMPs expression, local inflammation and plaque vulnerability, leading to significant reduction of plaque disruption in a rabbit model of vulnerable plaque. These effects were similar to a large dose of simvastatin and independent of serum lipid levels. The major mechanism underlying these effects involves downregualted expression of MMP2 and MMP9 and upregulated expression of collagen I in macrophages. Thus, doxycycline administration may provide a novel approach for the treatment of vulnerable atherosclerotic plaques.

## Supporting Information

Figure S1 **Relative mRNA expression of the inflammatory markers in the aortic plaque in three groups of rabbits.** Panel A, B, C, D, E, F, G and H depict the relative mRNA expression of the inflammatory markers in the aortic plaque in three groups of rabbits. Group D: doxycycline-treated group; Group S: simvastatin-treated group; Control: control group. *P<0.05, vs. Control group.
(TIF)

Figure S2 **Relative mRNA expression of the inflammatory markers in macrophages receiving different treatments.** Panel A, B and C depict the relative mRNA expression of MMP-2, MMP-9 and Collagen I in macrophages receiving doxycycline, simvastatin or no treatment. Group D: doxycycline-treated group; Group S: simvastatin-treated group; Control: control group. *P<0.05, vs. Control group. **P<0.01, vs. Control group. ***P<0.001, vs. Control group.
(TIF)
