# Supplementary figures and images for: Doxycycline Stabilizes Vulnerable Plaque via Inhibiting Matrix Metalloproteinases and Attenuating Inflammation in Rabbits
Source: PLoS One. 2012 Jun 21;7(6):e39695. doi: 10.1371/journal.pone.0039695 (PMC3380900; doi:10.1371/journal.pone.0039695)

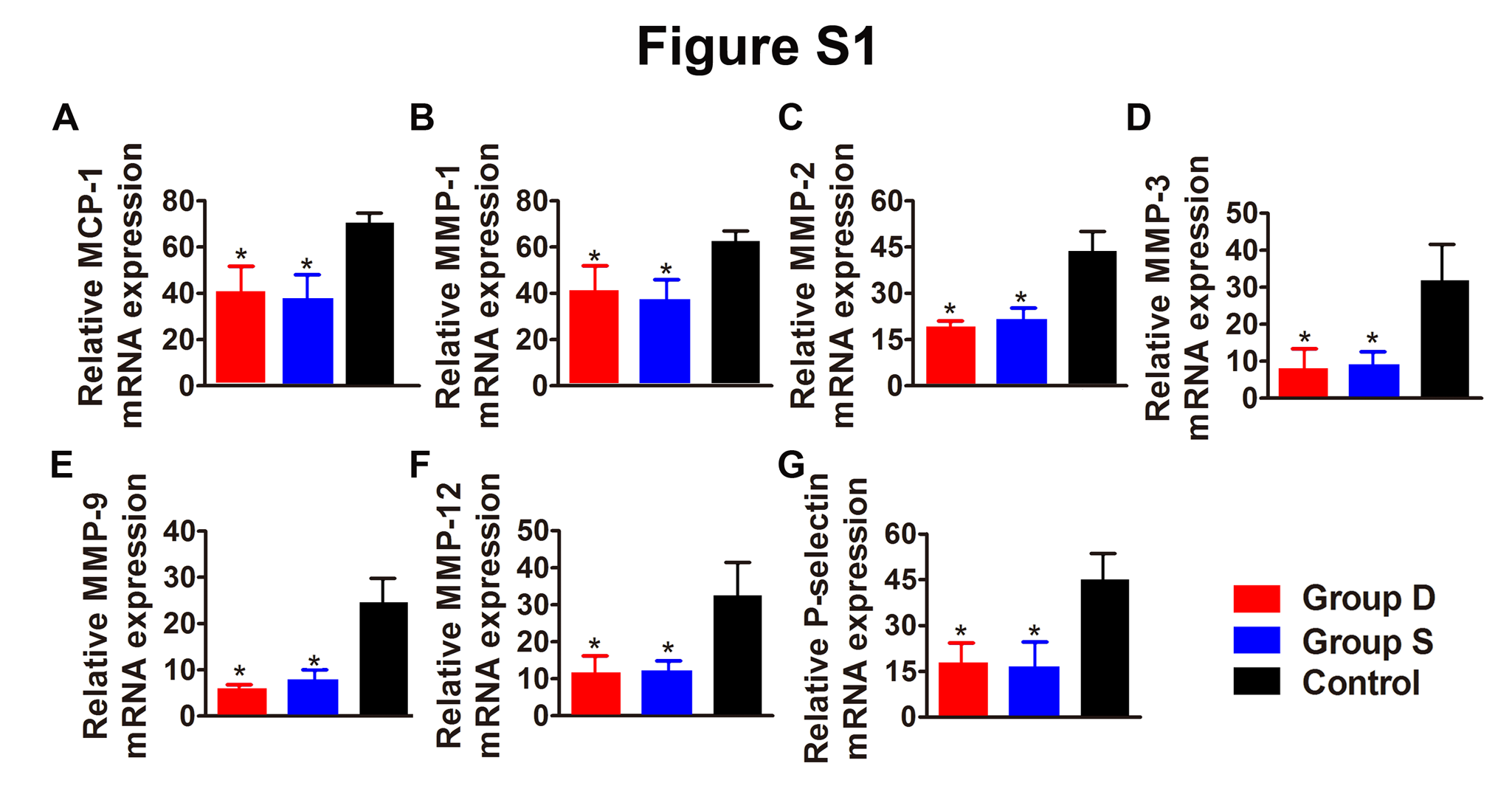

Supplement: Figure S1 — Relative mRNA expression of the inflammatory markers in the aortic plaque in three groups of rabbits. Panel A, B, C, D, E, F, G and H depict the relative mRNA expression of the inflammatory markers in the aortic plaque in three groups of rabbits. Group D: doxycycline-treated group; Group S: simvastatin-treated group; Control: control group. *P<0.05, vs. Control group. (TIF) [file pone.0039695.s001.tif]

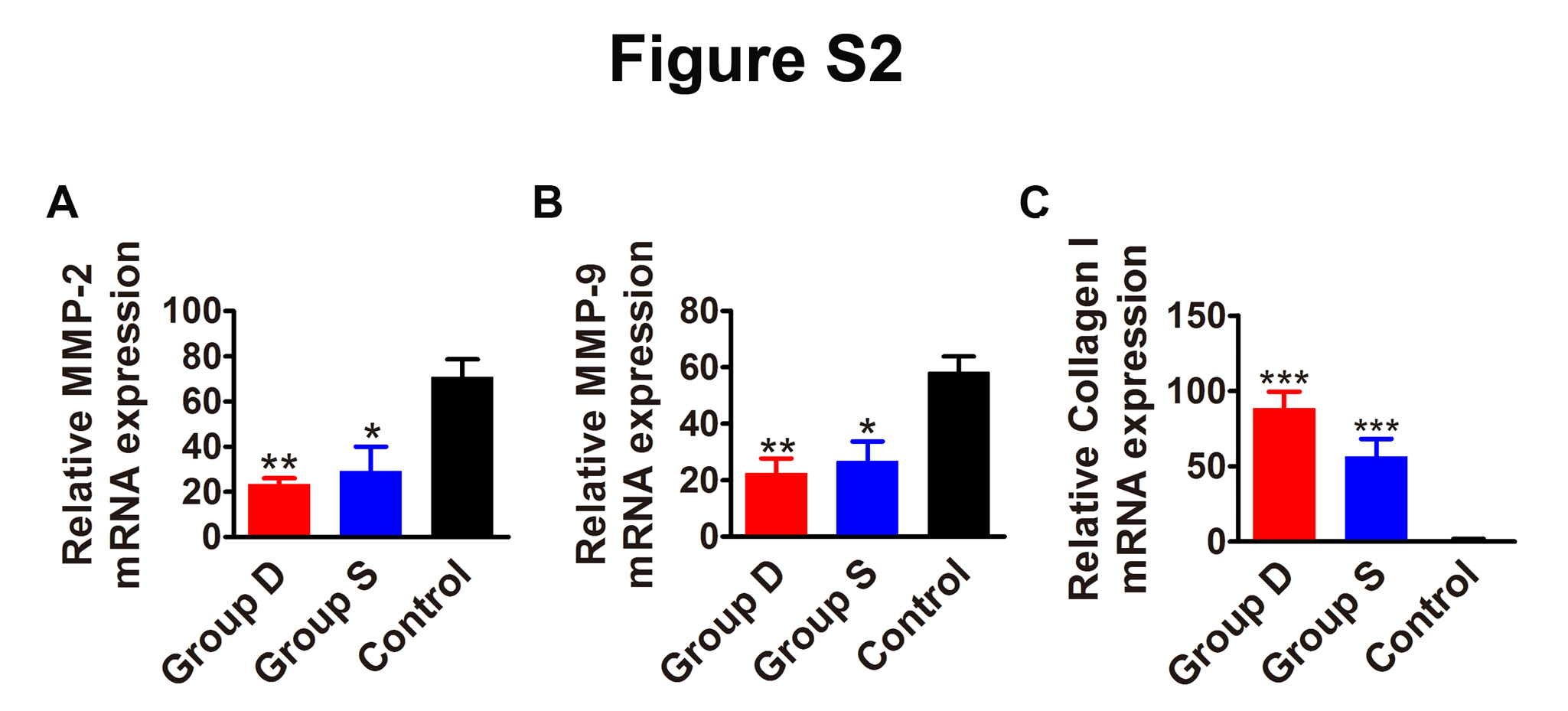

Supplement: Figure S2 — Relative mRNA expression of the inflammatory markers in macrophages receiving different treatments. Panel A, B and C depict the relative mRNA expression of MMP-2, MMP-9 and Collagen I in macrophages receiving doxycycline, simvastatin or no treatment. Group D: doxycycline-treated group; Group S: simvastatin-treated group; Control: control group. *P<0.05, vs. Control group. **P<0.01, vs. Control group. ***P<0.001, vs. Control group. (TIF) [file pone.0039695.s002.tif]
